# Two previously unknown *Phytophthora* species associated with brown rot of Pomelo (*Citrus grandis*) fruits in Vietnam

**DOI:** 10.1371/journal.pone.0172085

**Published:** 2017-02-16

**Authors:** Ivana Puglisi, Alessandro De Patrizio, Leonardo Schena, Thomas Jung, Maria Evoli, Antonella Pane, Nguyen Van Hoa, Mai Van Tri, Sandra Wright, Mauritz Ramstedt, Christer Olsson, Roberto Faedda, Gaetano Magnano di San Lio, Santa Olga Cacciola

**Affiliations:** 1 Department of Agriculture, Food and Environment, University of Catania, Catania, Italy; 2 Dipartimento di Agraria, Università Mediterranea di Reggio Calabria, Feo di Vito, Reggio Calabria, Italy; 3 Phytophthora Research Center Mendel University, Zemedelska 1, 613 00 Brno, Czech Republic; 4 Phytophthora Research and Consultancy, Am Rain 9, Nußdorf, Germany; 5 Southern Horticultural Research Institute, My Tho, Tien Giang, Vietnam; 6 Department of Electronics, Mathematics and Natural Sciences, University of Gävle, Gävle, Sweden; 7 Department of Forest Mycology and Plant Pathology, Swedish Agricultural University (SLU), Uppsala, Sweden; 8 Department of Biological and Environmental Sciences, Gothenburg University, Gothenburg, Sweden; Universita degli Studi di Pisa, ITALY

## Abstract

Two distinct *Phytophthora* taxa were found to be associated with brown rot of pomelo (*Citrus grandis*), a new disease of this ancestral *Citrus* species, in the Vinh Long province, Mekong River Delta area, southern Vietnam. On the basis of morphological characters and using the ITS1-5.8S-ITS2 region of the rDNA and the cytochrome oxidase subunit 1 (COI) as barcode genes, one of the two taxa was provisionally named as *Phytophthora* sp. prodigiosa, being closely related to but distinct from *P*. *insolita*, a species in *Phytophthora* Clade 9, while the other one, was closely related to but distinct from the Clade 2 species *P*. *meadii* and was informally designated as *Phytophthora* sp. mekongensis. Isolates of *P*. sp. prodigiosa and *P*. sp. mekongensis were also obtained from necrotic fibrous roots of Volkamer lemon (*C*. *volkameriana*) rootstocks grafted with ‘King’ mandarin (*Citrus nobilis*) and from trees of pomelo, respectively, in other provinces of the Mekong River Delta, indicating a widespread occurrence of both *Phytophthora* species in this citrus-growing area. Koch’s postulates were fulfilled via pathogenicity tests on fruits of various *Citrus* species, including pomelo, grapefruit (*Citrus* x *paradisi*), sweet orange (*Citrus* x *sinensis*) and bergamot (*Citrus* x *bergamia*) as well as on the rootstock of 2-year-old trees of pomelo and sweet orange on ‘Carrizo’ citrange (*C*. *sinensis* ‘Washington Navel’ x *Poncirus trifoliata*). This is the first report of a *Phytophthora* species from Clade 2 other than *P*. *citricola* and *P*. *citrophthora* as causal agent of fruit brown rot of *Citrus* worldwide and the first report of *P*. *insolita* complex in Vietnam. Results indicate that likely Vietnam is still an unexplored reservoir of *Phytophthora* diversity.

## Introduction

Pomelo [*Citrus grandis* (L.) Osbeck, syn. *C*. *maxima* (Burr.) Merr.], also known as pummelo, pommelo, shaddock or lusho fruit, is native to South-East Asia where it is very popular as a fruit crop and has an ancient history of cultivation and use dating back at least 4,000 years. Pomelo cultivars vary in size, shape and flesh color of the fruit. They hybridize freely among themselves and with other *Citrus* species, and numerous cultivars and local selections have been developed as a result. Pomelo is almost exclusively produced for the fresh fruit market; it has a very thick rind, which is an advantage for long term storage and transport, and reaches 10–30 cm in width and up to 9–10 kg in weight. It is, therefore, the largest of all citrus fruits, even larger than grapefruit (*Citrus* x *paradisi*), considered a hybrid of pomelo. Together with mandarin (*C*. *reticulata*) and citron (*C*. *medica*), pomelo is considered one of the few ‘true’ ancestral species from which all other cultivated citrus originated by hybridization [1]. Different from other citrus species, which grow and produce better in Mediterranean and subtropical climates, pomelo prefers hot and humid tropical climates. Although the origins of pomelo are debated, the islands of Fiji and Tonga, Malaysia and Thailand are considered the most probable centers of origin of pomelo, as inferred from the great genetic diversity of native germplasm [2]. As a matter of fact, in the Fiji islands pomelo grows wild on riverbanks.

In Vietnam, about 107,000 ha are cultivated with citrus and over 8,000 ha are planted with pomelo. The most important pomelo production areas are in the south because of the favorable tropical climate, but there are also minor production areas in the north of the country. The Vinh Long province, in the Mekong River Delta, is the leading growing area and here pomelo is produced for both domestic consumption and export and it is normally propagated by air layering (marcotting) or by grafting on Volkamer lemon (*C*. *volkameriana*). This rootstock tolerates root asphyxiation as a result of flooding or poor soil drainage but is susceptible to Phytophthora gummosis and root rot [3, 4].

*Phytophthora* species have been reported to cause diseases in a wide range of plant species in Vietnam, including major crops such as tomato, potato, citrus, pineapple, plum, black pepper, rubber, durian and jackfruit [5, 6]. However, in most cases, identification was based on disease symptoms and morphological characteristics of isolates. Phytophthora diseases of citrus have been studied only sporadically in Vietnam and studies have often been limited to surveys of disease incidence and severity.

In December 2012, during a survey aimed at identifying *Phytophthora* species infecting tropical fruit crops in southern Vietnam, fruits of pomelo with typical symptoms of Phytophthora brown rot were observed in commercial groves in Tam Bin, Vinh Long Province. Symptomatic fruits showed a rind decay in which the affected area was light brown, but tended to remain firm and leathery; white mycelium formed on the rind surface in humid conditions. An additional symptom was a characteristic pungent smell. Most symptomatic fruits were in the lower part of the tree canopy, close to the ground, and on the sampling data corresponded to about 10% of the total fruit production in the groves. They readily dropped off and fell to the ground.

The objectives of this study were to determine the etiology of this new disease, to characterize the *Phytophthora* isolates recovered from symptomatic pomelo fruits sourced in Tam Bin and compare these isolates with similar *Phytophthora* isolates recovered from necrotic roots of asymptomatic citrus trees in other provinces of the Mekong River Delta area.

## Materials and methods

### Plant material and isolation method

The study was carried out on private lands and the owner of the lands gave permission to conduct the study on this site. The study did not involve endangered or protected species. Symptomatic pomelo fruits were picked up from both the canopy and the soil under the tree canopy in five commercial groves in Tam Bin, Vinh Long Province. Fruits were washed carefully with running tap water, blotted dry, surface sterilized using household bleach (NaOCl, 1% available chlorine) for 1–2 min and rinsed in sterile distilled water. Small (3–5 mm) pieces of rind were cut out from the margin of brown lesions and plated onto BNPRA-HMI selective agar medium (10 ppm Benomyl, 25 ppm Nystatin, 25 ppm Pentachloronitrobenzene, 10 ppm Rifampicin, 500 ppm Ampicillin, 50 ppm Hymexazol and Potato Dextrose Agar (PDA) as basal medium) [7]. Petri dishes were incubated at 24°C for 3–6 days in the dark. Pure cultures of *Phytophthora* were obtained by transferring single hyphal tips under the stereomicroscope onto PDA (BD Difco, Italy). Necrotic fibrous roots were sampled from five asymptomatic ‘King’ mandarin trees grafted on Volkamer lemon rootstock and 5 pomelo trees obtained by marcotting, in two distinct groves in the Dong Thap and Ben Tre provinces, respectively. No one of the 10 above-mentioned trees showed symptoms in the canopy notwithstanding the presence of necrotic roots. Roots were washed free of soil under running tap water, blotted dry, cut into short segments (3–5 mm) and plated onto BNPRA-HMI agar in Petri dishes. Petri dishes were incubated at 24°C for 3–6 days in the dark. Subcultures of *Phytophthora* colonies emerging from root pieces were obtained by transferring single hyphal tips under the stereomicroscope onto PDA and V8 vegetable juice agar (V8A, Campbell Foods, Belgium).

### Morphological and cultural characteristics

Colony types, growth rates at different temperatures and cardinal temperatures for growth, and morphology of sporangia, chlamydospores and hyphal swellings were determined, on both V8A and PDA following published methods [8, 9, 10]. Three 90 mm Petri dishes of PDA and V8A (20 mL each) were inoculated centrally with mycelia agar plugs taken from an actively growing colony kept at 24°C. Inoculated dishes were incubated in the dark between 4 and 40°C with 4°C intervals and measurements of mycelial growth were made by taking two orthogonal diameters of the developed colonies before they reached the edge of the Petri dishes. The experiment was repeated twice. Sporangia, hyphal swellings and chlamydospores were examined after two weeks of growth at 24°C on the two agar media used for the growth tests. Pieces from the margin of actively growing cultures on V8A were overlaid with non-sterile soil filtrate, according to the method of Jung et al. [11], to stimulate sporangium formation. Dimensions of 50 mature sporangia of each isolate, chosen at random, and their characteristic features were determined using a light microscope at ×400 and ×1000 magnifications. For microscopical examinations slides were mounted in distilled water.

Mating type tests were carried out on carrot agar (CA) and clarified V8 juice agar (cV8A), prepared as described by Erwin and Ribeiro [12], and following the method reported by Brasier et al. [8]. A1 and A2 tester isolates were sourced from the Di3A culture collection and they were Pf2b and CH230 for *P*. *nicotianae*, isolated from pomelo in Vietnam [13], and AX1aC and CC1aL for *P*. *citrophthora*, isolated from the rhizosphere soil of *Citrus aurantium* trees in Italy [14].

For both new *Phytophthora* species a detailed description of morphological characteristics, morphometric data, cardinal temperatures and growth rates, and their comparison with closely related species together with a multigene phylogeny will be presented in a separate publication.

### PCR amplification and sequencing

Genomic DNA was extracted from 10 mg of fresh mycelium collected from PDA plates using the Power Plant Pro DNA Isolation Kit (MO BIO Laboratories, USA). Quality and quantity of extracted DNA samples were evaluated using a DNA Quant-it assay kit (Molecular Probes, Carlsbad, CA, USA) and by electrophoresis in 1% agarose gels containing SYBR® Safe (Invitrogen, Life Technologies, USA) DNA gel stain.

The internal transcribed spacer (ITS) region of the rDNA was amplified and sequenced for all *Phytophthora* isolates using primers ITS6 and ITS4 as described by Cooke et al. [15]. PCR consisted of 95°C for 5 min, 35cycles of 95°C for 30 sec, 55°C for 30 sec, 72°C for 30 s and a final cycle of 72°C for 10 min.

The oomycete-specific primers OomCoxILevup and Fm85mod were used to amplify the fragment of cytochrome oxidase subunit 1gene (COI) from mitochondrial DNA as described in Robideau et al. [16]. PCR consisted of 95°C for 2 min followed by 35 cycles of 95°C for 1 min, 55°C for 1 min, 72°C for 1 min and a final extension at 72°C for 10 min.

Amplicons were analyzed by electrophoresis, visualized in a 1.5% agarose gel and photographs were scanned through a Gel Doc System (Biorad). PCR products were quantified using a DNA Quant-it assay kit (Molecular Probes, Carlsbad, CA, USA), purified using the ExoSAP-ITkit for PCR Product Cleanup (Affymetrix, UK) and sequenced using an external sequencing service (BMR-genomic, Italy).

### Sequence analysis of ITS and COI regions

The Basic Local Alignment Search Tool (BLASTN) was used to compare sequences from the National Center for Biotechnology Information (NCBI) (http://www.ncbi.nlm.nih.gov) and the *Phytophthora* Database (www.phytophthoradb.org) [17, 18].

Sequences of all Vietnamese isolates were submitted to GenBank and the corresponding accession numbers are listed in (S1 Table). ITS and COI sequences of the Vietnamese isolates and from representative isolates of Clades 2 and 9 retrieved by Robideau et al. [16] were used in the phylogenetic analysis to determine the phylogenetic position and taxonomic status of the Vietnamese isolates (S2 Table). ITS and COI sequences were separately aligned and compared with the reference sequences from Robideau et al. [16] using the software MUSCLE (http://www.ebi.ac.uk/ Tools/msa/muscle/) and MEGA6 [19, 20]. Phylogeny reconstruction was performed with MEGA6 [19, 20] using Maximum Likelihood method and the Tamura-Nei model [21]. Bootstrap values were obtained from 1000 repetitions.

### Pathogenicity tests

To test the pathogenicity of *Phytophthora* species recovered from pomelo fruits and fine roots of pomelo and “King” mandarin trees, three representative isolates from fruits, PF6a2, PF6f2 and PF6e, as well as two representative isolates from roots, Pr3 from pomelo and Pr1 from ‘King’ mandarin, were inoculated singly on mature fruits of pomelo ‘Chandler’, grapefruit ‘Marsh Seedless’, sweet orange (*Citrus* x *sinensis*) ‘Tarocco Ippolito’ and ‘Valencia late’, and bergamot (*Citrus* x *bergamia*) ‘Castagnaro’. Rind cores were cut aseptically from fruits (5 fruits of each citrus species per isolate) with a cork-borer (3 mm diameter) and 3 mm agar plugs from the edge of actively growing cultures were placed in the holes. Sterile agar plugs were used for control fruits. The cores were replaced and sealed with adhesive tape. Fruits were incubated in plastic bags at 24–26°C in darkness. In two additional separate experiments, fruits of the same citrus varieties were inoculated without wounding using two different methods. In the first experiment 3 mm agar plugs from actively growing cultures on V8A were placed on the rind of fruits (5 fruits of each citrus variety per isolate) in an equatorial position without wounding. Plain agar blocks were included as controls. Fruits were incubated in plastic bags at 24–26°C in darkness. Distilled water was nebulized, after inoculation, into the bags after the inoculation to reach a high relative humidity and prevent dehydration of agar blocks.

In the second experiment, a zoospore suspension was produced using the protocol described by Scanu & Webber [22]. Discs, 1 cm in diameter, were cut from the edges of colonies growing on CA and floated in plates containing sterile Petri solution for 48 h at 20°C under continuous daylight. The Petri solution was then replaced with sterile water and plates incubated for a further 48–72 h. Once sporangia were plentiful on the discs of mycelia, the plates were kept at 4°C for 1 h and returned to room temperature to induce zoospore release. Zoospore concentration was determined using a haemocytometer and its concentration was adjusted to 104 zoospores ml-1. A drop (200 μl) of the suspension was pipetted onto the non-wounded rind of fruits (5 fruits of each citrus variety per isolate) in a polar position near the rosette. Sterile distilled water was included as a control. Fruits were incubated in plastic bags at 24–26°C in darkness. Distilled water was nebulized into the bags to reach a high relative humidity and prevent dehydration of the inoculum. In all three experiments the size of each lesion induced by artificial inoculation was determined as the mean of two orthogonal diameters, six days after the inoculation.

The five *Phytophthora* isolates were also used to inoculate 2-year-old potted trees of pomelo ‘Chandler’ and sweet orange ‘Lane Late’ grafted on ‘Carrizo’ citrange (*C*. *sinensis*‘ Washington Navel’ x *Poncirus trifoliata*) rootstock, which is known a *Phytophthora*-resistant rootstock [3, 4, 23]. Trees (5 trees per treatment) were inoculated on the twigs (3 twigs, 1.5–2 cm in diameter, per tree) by cutting bark disks with a cork-borer (diameter 3 mm) and inserting an agar plug from actively growing cultures, with mycelium inside facing the wood. Trees were inoculated also on the basal portion of the stem, on the rootstock, at two heights from the ground (5 and 15 cm). Trees inoculated with plain agar blocks were included as controls. The removed bark disk was replaced, and the wound was then sealed with Parafilm®. Trees were grown in a greenhouse at a temperature ranging from 22 to 32°C. After inoculation, twigs and stems were inspected daily for symptoms. Thirty days after inoculation, the bark around the inoculation points of both twigs and stem was removed and the length of discoloration on the wood was measured. The measurement of the lesion included the diameter (5mm) of the hole made with the cork borer. The test was repeated twice.

### Statistical analyses

Pathogenicity data were analyzed by one-way analysis of variance (ANOVA) using Tukey’s HSD test (Honestly Significant Difference) as a post-hoc test (XLSTAT 2008 software). Differences at P ≤ 0.05 were considered significant.

## Results

### Morphological identification of *Phytophthora* isolates

**Fig 1:**
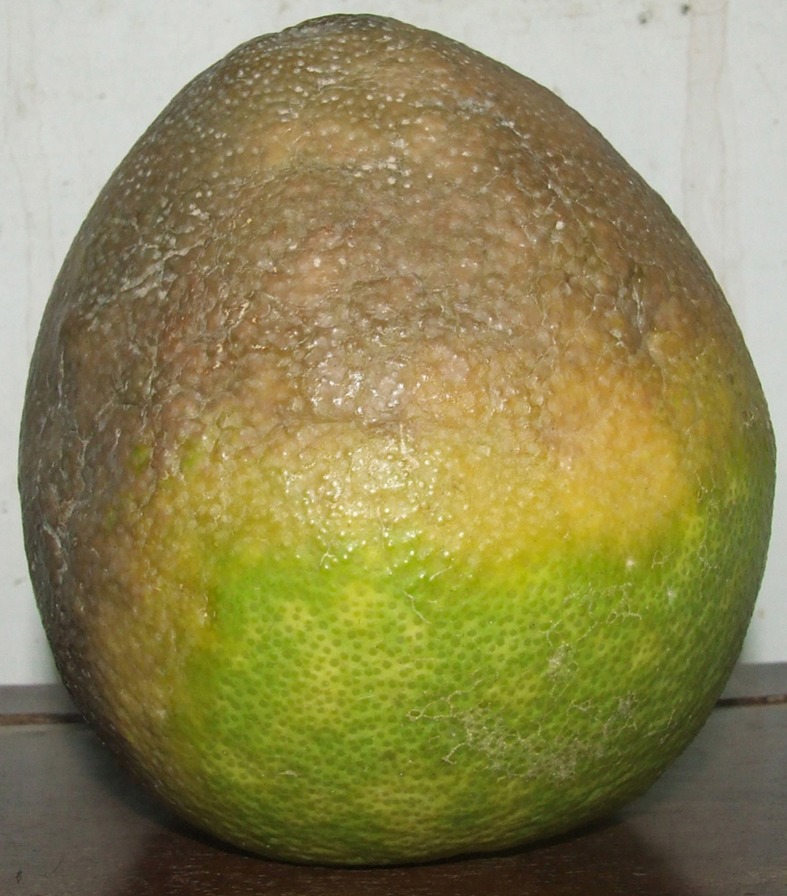
Fruit of pomelo (*Citrus grandis*) with symptoms of brown rot caused by mixed infections of *P*. sp. prodigiosa and *P*. sp. mekongensis from Vietnam.

*Phytophthora* isolates were consistently obtained from symptomatic pomelo fruits (Fig 1) sampled in citrus groves at Tam Bin. A total of 89 *Phytophthora* isolates were characterized in this study: 38 isolates were obtained from distinct pomelo fruits harvested from the tree canopy and 41 from distinct pomelo fruits collected from the soil under the tree canopy; the other 10 isolates were obtained in commercial citrus groves from fine roots of ‘King’ mandarin trees grafted on Volkamer lemon (*C*. *volkameriana*) rootstock in the Dong Thap province (five isolates) and marcotted pomelo trees in the Ben Tre province (five isolates), respectively. Representative isolates of three distinct *Phytophthora* morphological groups recovered from pomelo fruits during the survey and separated on the basis of colony morphology (isolates PF6a2 = CBS 135136; PF6f2 = CBS 135137 and PF6e = CBS 135138) were deposited as living cultures in the collection of Centraalbureau voor Schimmelcultures (CBS), Fungal Biodiversity Centre, Utrecht, The Netherlands.

**Fig 2:**
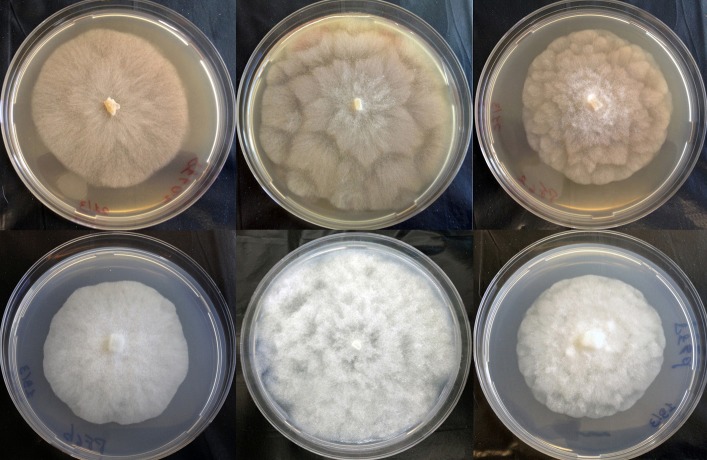
Five-day-old colonies of isolates PF6a2 and PF6f2 of *P*. sp. mekongensis and isolate PF6e of *P*. sp. prodigiosa (from left to right) on V8 juice agar (top) and potato-dextrose agar (bottom).

On the basis of colony growth patterns and characteristic features of morphological structures, the isolates were first grouped in three distinct morphotypes. The first and second type were represented by isolates PF6a2 and PF6f2, respectively, and are informally designated here as *P*. sp. mekongensis, while the third type was represented by isolate PF6e and is informally designated here *P*. sp. prodigiosa. On V8A the colony pattern of morphotype 1 (isolate PF6a2) was stellate while morphotypes 2 and 3 (isolates PF6f2 and PF6e) formed rosaceous colonies. On PDA the colony pattern of morphotypes 1 and 2 (isolates PF6a2 and PF6f2) was stellate, while morphotype 3 (isolate PF6) formed rosaceous colonies (Fig 2).

**Fig 3:**
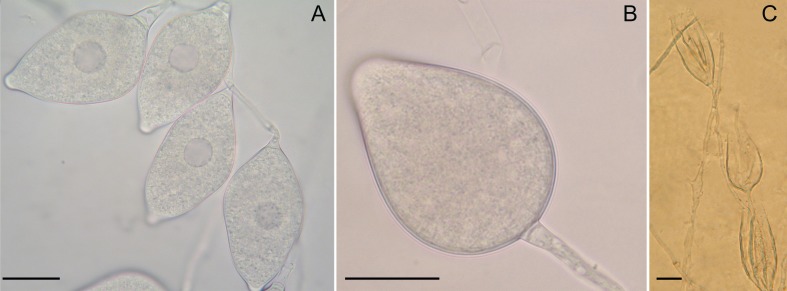
Morphology of vegetative structures of *Phytophthora* isolates. A. Cluster of fusiform, papillate sporangia of *P*. sp. mekongensis. B. Obpyriform, persistent, nonpapillate sporangium of *P*. sp. prodigiosa. C. Internal and external extended proliferations of persistent sporangia of *P*. sp. prodigiosa produced in water. (Scale bar = 20 μm).

The morphology of sporangia produced by morphotypes 1 and 2 overlapped. Both morphotypes produced ovoid-obpyriform, ellipsoid to fusiform, papillate, frequently bi-papillate and bilobed, often caducous (pedicel length 5 to 15 μm) sporangia with an average size of 35 x 24 μm (25–50 x 20–36 μm) and a mean l/b ratio of 1.5, with a conspicuous basal plug at the point where the pedicel attaches to the sporangium (Fig 3). Morphotype 2 often produced trilobed sporangia. The minimum, optimum and maximum temperatures for growth of the two morphotypes were 12, 28 and 36°C and 8, 24–28 and 32°C, respectively.

**Fig 4:**
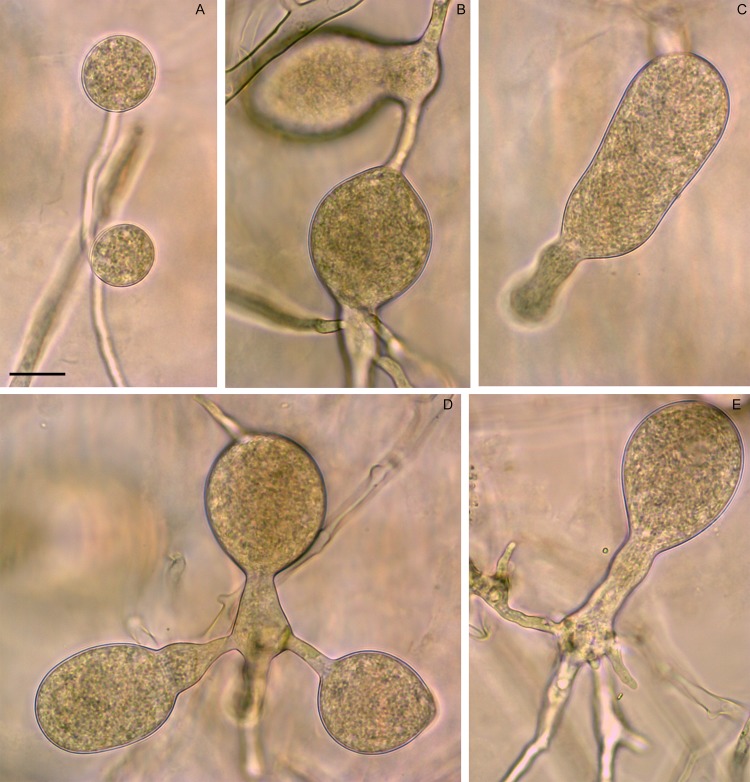
Chlamydospores and hyphal swellings produced by *P*. sp. prodigiosa. (A). Globose, small, sessile chlamydospores on V8A, attached laterally to the hypha and with a thin wall. (B-E) Hyphal swellings of *P*. sp. prodigiosa on V8A with bizarre shapes (Scale bar = 20 μm).

Morphotype 3 produced pyriform to obpyriform, ovoid, non-papillate, persistent sporangia that proliferated internally in a nested or extended way (Fig 3). Average dimensions were 45 x 32 μm (30 to 50 x 19 to 34 μm) with a mean l/b ratio of 1.4. Chlamydospores of variable size (20 to 48 μm), globose to obpyriform, sometimes attached laterally to the hypha, as well as catenulate, elongated to globose hyphal swellings, often with a bizarre shape, were abundantly formed on V8A (Fig 4). No sexual structure was observed in single culture. The minimum, optimum and maximum temperatures for growth were 12, 32 and 36°C.

None of the isolates of morphotypes 1, 2 and 3 produced gametangia in dual cultures with A1 and A2 mating type tester strains of *P*. *nicotianae* and isolates of *P*. *citrophthora* from citrus.

All three morphotypes were isolated together from all pomelo fruits collected from the soil, with the prevalence of morphotype 2 (about 80% of the overall number of isolates), whereas brown rot of pomelo fruits harvested from the tree canopy was exclusively caused by mixed infections of morphotypes 1 and 2. During the survey, isolates of morphotype 3 were also obtained from necrotic fibrous roots of ‘King mandarin’ trees in the Dong Thap province while isolates of morphotype 2 were obtained from necrotic fibrous roots of pomelo trees in the Ben Tre province. The complete list of isolates characterized in this study is given in S1 Table.

### Molecular identification of *Phytophthora* isolates

A conventional sequence analysis of the internal transcribed spacer (ITS) region of ribosomal DNA and the fragment of cytochrome oxidase subunit 1 gene (COI) was performed to identify the species. Representative sequences of each *Phytophthora* isolate were submitted to GenBank under the following accession numbers: KC875838 (ITS isolate PF6a2, *P*. sp. mekongensis), KT366920 (COI isolate PF6a2, *P*. sp. mekongensis); KC875839 and KM501564 (ITS isolates PF6f2 and Pr3, *P*. sp. mekongensis, respectively); KT366919 and KU640394 (COI isolate PF6f2 and Pr3, *P*. sp. mekongensis); KC875840 and KM501564 (ITS isolates PF6e and Pr1, *P*. sp. prodigiosa); KT366918 and KU363433 (COI isolate PF6e and Pr1, *P*. sp. prodigiosa) (Table 1). Both ITS and COI sequences of morphotype 2 isolates PF6f2 from fruit rot and Pr3 from a necrotic fibrous root were 100% identical. Similarly, sequences of ITS and COI regions of morphotype 3 isolate PF6e from fruit showed 100% homology with the corresponding sequences of morphotype 3 isolate Pr1 from a necrotic fibrous root. Furthermore, comparing ITS and COI sequences of morphotype 1 isolate PF6a2 and morphotype 2 isolate PF6f2, 99.74% (8 bp difference) and 100% identity was demonstrated for ITS and COI, respectively, despite the morphological differences between these two isolates. A BLAST search for the ITS sequences of the representative isolates PF6f2 and Pr3 (morphotype 2), PF6a2 (morphotype 1), and PF6e and Pr1 (morphotype 3) using the Phytophthora database (www.phytophthoradb.org), showed 99% identity to *P*. *meadii* (GenBank no. GU259182), *P*. *colocasiae* (GenBank no. GU258988) and *P*. *insolita* (GenBank no. GU258764), respectively.

**Table 1:** *Phytophthora* species sampled in the Mekong River delta (Vietnam), source, host, location of recovery and GenBank accession numbers of ITS and COI sequences of representative isolates.

*Phytophthora* species	Number of isolates	Representative isolate	Source	Host	Geographic origin (province)	ITS	COI
*P*. sp. mekongensis	34	PF6a2	Fruit rot	Pomelo	Vĩnh Long	KC875838	KT366920
*P*. sp. mekongensis	35	PF6f2	Fruit rot	Pomelo	Vĩnh Long	KC875839	KT366919
*P*. sp. mekongensis	5	Pr3	Root rot	Pomelo	Ben Tre	KM501564	KU640394
*P*. sp. Prodigiosa	10	PF6e	Fruit rot	Pomelo	Vĩnh Long	KC875840	KT366918
*P*. sp. Prodigiosa	5	Pr1	Root rot	Mandarin/ Volkamer lemon	Dong Thap	KM501564	KU363433

**Fig 5:**
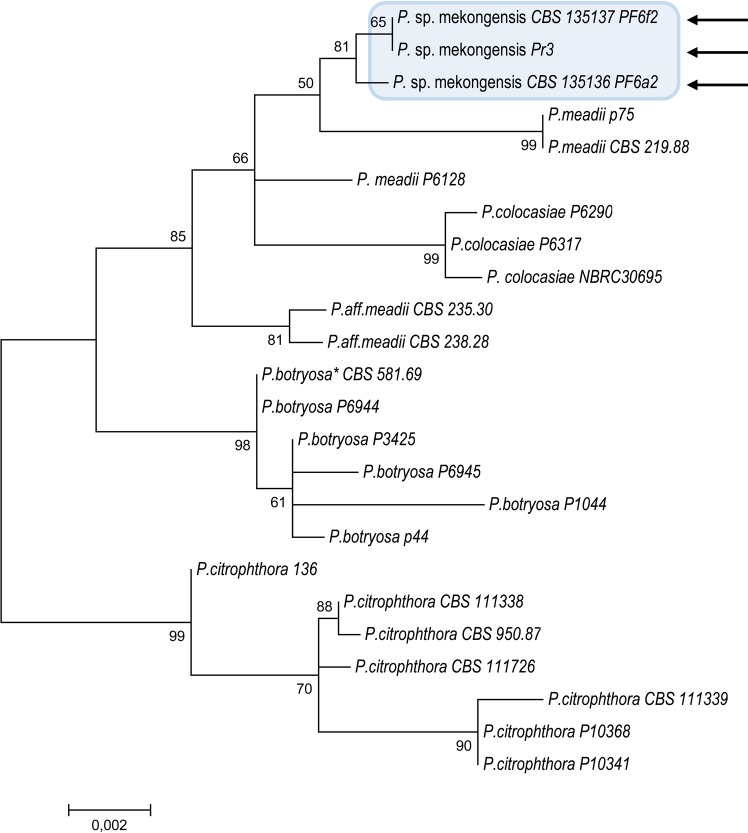
Molecular phylogenetic analysis of the combined data set ITS and COI among isolates Pf6f2, Pr3 and Pf6a2 of *P*. sp. mekongensis (indicated by arrows) within *Phytophthora* major Clade 2 by Maximum Likelihood method, based on the Tamura-Nei model. The percentage of trees in which the associated taxa clustered together is shown next to the branches. Initial trees for the heuristic search were obtained by applying the Neighbor-Joining method to a matrix of pairwise distances estimated using the Maximum Composite Likelihood (MCL) approach. The tree was drawn to scale, with branch lengths measured in the number of substitutions per site. Bootstrap values were obtained from 1000 replications. Asterisk (*) indicates the ex-type isolate.

Fig 5 shows the phylogenetic position of isolates PF6a2 (morphotype 1) and PF6f2 and Pr3 (morphotype 2) using a maximum likelihood analysis of a combined data set of ITS and COI sequences of these three isolates and other *Phytophthora* species from Clade 2 (S2 Table), retrieved from Robideau et al. [16]. Both morphotypes resided in Clade 2 and were closely related to, but distinct from, *P*. *meadii* and *P*. *colocasiae*. A similar tree topology was also observed when the two regions were analyzed separately (data not shown). On the basis of the phylogenetic analysis of ITS and COI sequences with 99.74% and 100% homology of the ITS and COI sequences, respectively, the morphotypes 1 and 2, represented by isolates PF6a2, PF6f2 and Pr3, are here provisionally designated as *Phytophthora* sp. mekongensis because these isolates were recovered in the region of the Mekong river delta.

**Fig 6:**
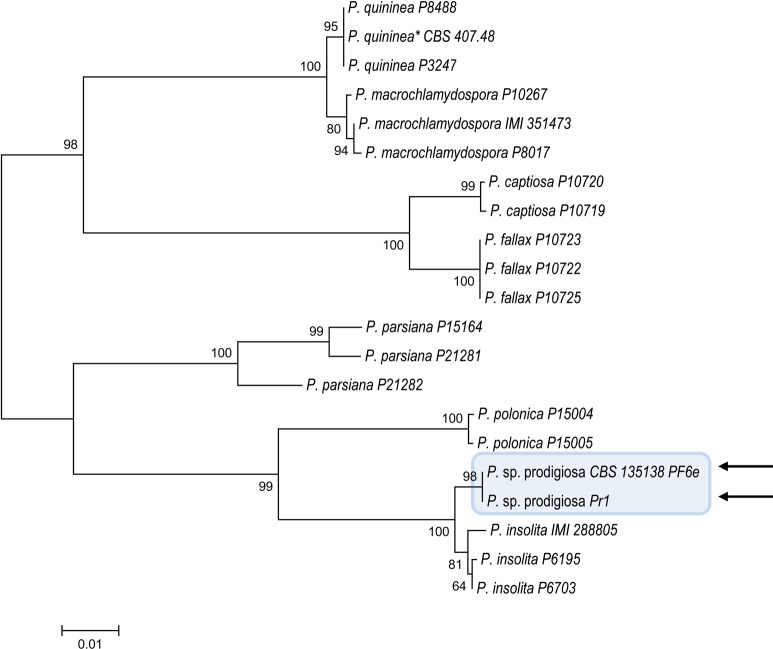
Molecular phylogenetic analysis of the combined data set ITS and COI among isolates PF6e and Pr1 of *P*. sp. prodigiosa (indicated by arrows) within *Phytophthora* major Clade 9 by Maximum Likelihood method, based on the Tamura-Nei model. The percentage of trees in which the associated taxa clustered together is shown next to the branches. Initial trees for the heuristic search were obtained by applying the Neighbor-Joining method to a matrix of pairwise distances estimated using the Maximum Composite Likelihood (MCL) approach. The tree was drawn to scale, with branch lengths measured in the number of substitutions per site. Bootstrap values were obtained from 1000 replications. Asterisk (*) indicates the ex-type isolate.

Results of phylogenetic analysis of the combined data set of ITS and COI sequences of morphotype 3 isolates PF6e and Pr1 and validated sequences of species in Clade 9 are shown in Fig 6. A similar tree topology was also observed when the two regions were analyzed separately (data not shown). Morphotype 3 isolates are here provisionally designated as *Phytophthora* sp. prodigiosa because of the bizarre and unusual shape of hyphal swellings although all 15 isolates included in this morphotype (Table 1) showed many morphological characteristics corresponding to the original description of *P*. *insolita* by Ann & Ko [12, 24, 25] and clustered with reliable sequences deposited with this name. However, DNA sequences of the ex-type isolate of this species are not available and alignments among sequences of isolates from pomelo and reference sequences of *P*. *insolita* deposited in databases suggest that this taxon is polyphyletic.

### Pathogenicity tests

Wound-inoculations and inoculations of non-wounded fruits, using both agar discs and zoospore suspension, produced symptoms of brown rot 5–6 days after inoculation with all three *Phytophthora* morphotypes. On fruits inoculated with *P*. sp. mekongensis isolates PF6a2, PF6f2 and Pr3 a dense layer of white mycelium developed on the lesions. The mean diameter of lesions induced by isolates of the three species on wounded and non-wounded fruits 6 days after inoculation is shown in Table 2. In all three experiments, the highest values were obtained for pomelo and grapefruit, indicating these were the most susceptible citrus species, while on bergamot fruits mean lesion size was lower compared to other tested citrus species. Moreover, in all experiments and for all tested citrus species the *P*. sp. prodigiosa isolates were less aggressive than the isolates of *P*. sp. mekongensis. Among the isolates of the latter species, PF6a2, representing morphotype 1, was the most virulent in all tests whereas isolates Pf6f2, representing morphotype 2, and Pr3 did not differ significantly. Also *P*. sp. prodigiosa isolates PF6e and Pr1, from fruit and root respectively, did not differ significantly in virulence. Control fruits showed no symptoms and the same *Phytophthora* taxa used to inoculate the fruits were re-isolated solely from symptomatic fruits.

**Table 2:** Area (cm^2^) of lesions caused by isolates PF6a2, PF6f2, and Pr3 of *P*. sp. mekongensis and isolates PF6e and Pr1 of *P*. sp. prodigiosa on fruits of various citrus species and cultivar inoculated with (A) agar plugs on wounded fruits, (B) agar plugs on unwounded fruits and (C) a drop (200 μl) of zoospore water-suspension on unwounded fruits, 6 days after inoculation.

			*Citrus* species and cultivar				
Inoculation method	*Phytophthora* sp.	Isolate code	Pomelo ‘Chandler’	Grapefruit ‘Marsh Seedless’	Sweet orange ‘Tarocco Ippolito’	Sweet orange ‘Valencia Late’	Bergamot ‘Castagnaro’
A	*P*. sp. mekongensis	PF6a2	67.9 a	62.2 ab	39.6 ef	41.8 df	26.4 g
		PF6f2	56.7 bc	45.3 d	36.3 f	38.5 ef	26.4 g
		Pr3	55.4 c	46.5 d	38.5 ef	36.3 f	26.4 g
	*P*. sp. prodigiosa	PF6e	47.8 d	41.8 def	27.3 g	28.3 g	24.6 g
		Pr1	44.2 de	38.5 ef	26.4 g	26.4 g	23.7 g
	Control		0 h	0 h	0 h	0 h	0 h
B	*P*. sp. mekongensis	PF6a2	50.2 a	44.2 ab	24.6 fg	28.3 efg	16.6 hi
		PF6f2	49.0 ab	38.5 bc	30.2 def	32.2 cde	22.9 gh
		Pr3	50.2 a	43.0 b	36.3 cd	30.2 def	22.1 ghi
	*P*. sp. prodigiosa	PF6e	12.6 lmn	10.7 lmn	9.1 n	9.6 m	7.1 n
		Pr1	15.9 ilm	10.7 lmn	11.9 ln	9.1 n	8.5 n
	Control		0 o	0 o	0 o	0 o	0 o
C	*P*. sp. mekongensis	PF6a2	56.7 a	50.2 ab	31.2 e	32.2 e	15.9 gh
		PF6f2	44.2 bc	40.7 cd	36.3 de	35.2 d	21.2 f
		Pr3	45.3 bc	43.0 c	36.3 de	32.2 e	22.1 f
	*P*. sp. prodigiosa	PF6e	24.6 f	23.7 f	10.2 hl	9.6 hl	7.1 l
		Pr1	23.7 f	22.1 fg	11.9 hl	9.6 hl	8.0 l
	Control		0 m	0 m	0 m	0 m	0 m

Each value represents the mean of five replicated values. In each experiment numbers followed by different letters are significantly different values of the lesions according to Tukey’s HSD test (P ≤ 0.05).

**Fig 7:**
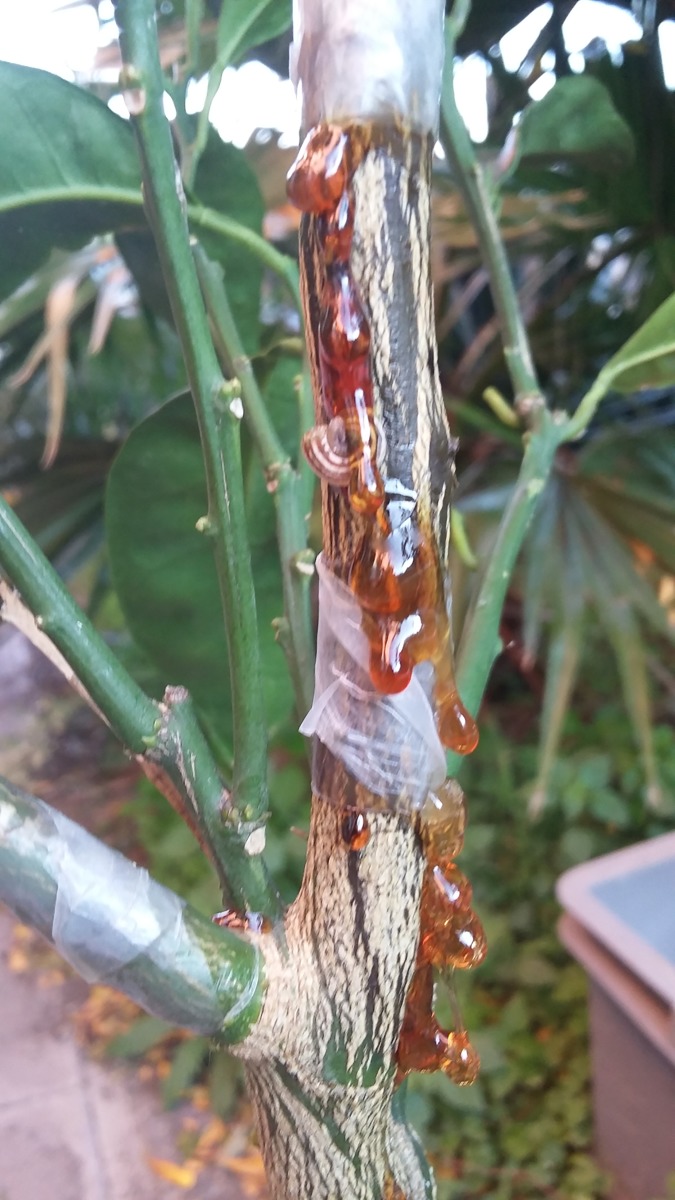
Gum exudate oozing from the twig of a sweet orange ‘Lane Late’ wound inoculated with isolate PF6f2 of *P*. sp. mekongensis from Vietnam, 12 days after inoculation.

The results of pathogenicity tests on potted citrus trees in three separate experiments were very similar and, hence, only results of a single experiment are reported. On twigs of pomelo ‘Chandler’ and sweet orange ‘Lane late’ inoculated with *P*. sp. mekongensis isolates PF6a2, PF6f2 and Pr3, gum exudates oozing profusely from the bark were first observed 7 days after the inoculation (Fig 7) while isolates of *P*. sp. prodigiosa did not induce gum exudation. Gum exudates on the stem of ‘Carrizo’ citrange rootstock were first observed two weeks after the inoculation and only on trees inoculated with *P*. sp. mekongensis isolates PF6f2 and Pr3. The mean length of cankers induced by the isolates on twigs and stems 30 days after inoculation is reported in Table 2. Isolates PF6f2 and Pr3 of *P*. sp. mekongensis were the most virulent on both twigs and stems. The mean length of cankers induced by these two isolates did not differ significantly whereas isolate PF6a2 was the least virulent among the isolates of this species. On twigs of susceptible citrus species, pomelo and sweet orange, it was more virulent than the isolates PF6e and Pr1 of *P*. sp. prodigiosa, but did not differ significantly from them in stem inoculations of the resistant citrange rootstock. Controls inoculated with sterile agar showed no symptoms. All inoculated *Phytophthora* taxa were re-isolated from inoculated symptomatic twigs and stems whereas no *Phytophthora* could be isolated from the controls.

## Discussion

Several species of *Phytophthora* are known to infect citrus worldwide, the most widespread being *P*. *nicotianae* from *Phytophthora* Clade 1 and *P*. *citrophthora* from Clade 2 [12, 23, 26, 27, 28]. *Phytophthora nicotianae*, which has an optimum growth temperature of 28–30°C, is more common in tropical and subtropical areas and causes foot rot, gummosis and root rot, but usually does not infect tissues far above the ground. *Phytophthora citrophthora*, which has a lower optimum temperature, causes gummosis and root rot in Mediterranean climates, where seasonal rainfall occurs during the cool winter months. This species also attacks aerial parts of the trees and is the most common causal agent of brown rot of citrus fruits in Mediterranean regions [29]. *Phytophthora palmivora* from Clade 4 is highly pathogenic on fibrous roots and is a common agent of fruit brown rot in various humid subtropical and tropical areas of the world [23]. All three species have been reported as citrus pathogens in Vietnam [30]. Other species, including *P*. *citricola* from Clade 2, *P*. *hibernalis* and *P*. *syringae* (both from Clade 8), have been reported worldwide to cause fruit brown rot occasionally and to a limited extent [23, 31].

*Phytophthora citrophthora*, a species from ITS Clade 2a [15, 32], was the species most frequently cited as a citrus pathogen in Vietnam. It was reported as causal agent of trunk gummosis and fruit rot of oranges [5]. *Phytophthora nicotianae*, which is more thermophilic than *P*. *citrophthora*, is another common citrus pathogen in Vietnam and was the prevalent species found in citrus groves in the Ben Tre and Tien Giang provinces of southern Vietnam [33]. *Phytophthora* species in Clade 2a closely related to *P*. *citrophthora* and already reported in Vietnam on other crops include *P*. *botryosa*, *P*. *colocasiae* and *P*. *meadii*. *Phytophthora botryosa* was first reported and described by Chee [34] and is known as causal agent of leaf fall and pod rot of rubber trees (*Hevea brasiliensis*) in several countries of south-east Asia, including Vietnam [5]. It also causes pod rot of cacao (*Theobroma cacao*) and was reported in China on taro (*Colocasia esculenta*) [12]. *Phytophthora colocasiae* was first described in 1900 as the causal agent of taro leaf blight in Indonesia and is considered to be primarily a foliar pathogen with a limited host range [12]. This pathogen is the major limiting factor for the cultivation of this important tropical storage food crop in south-east Asia and the Pacific and Caribbean regions [35], although an Asiatic origin seems probable [36] as Asia is the center of origin of many wild and cultivated species of taro [37]. *Phytophthora meadii* was first described in 1918 as a pathogen of rubber tree (*Hevea brasiliensis*) and, like *P*. *colocasiae*, is considered a species with a limited natural host range [12]. Very recently, a phylotype associated to *P*. *meadii* was reported for the first time in Europe in the rhizosphere of ornamental and citrus plants using an amplicon metagenomics approach based on 454 pyrosequencing and cloning/Sanger sequencing [38, 39].

*Phytophthora colocasiae* and *P*. *meadii* have been previously reported in Vietnam on several hosts, but not on citrus [30, 40]. In detail, morphological and molecular boundaries between *P*. *meadii*, *P*. *colocasiae* and *P*. *botryosa*, and between these three species and other closely related species in Clade 2 such as, *P*. *citrophthora*, *P*. *himalsilva* as well as the recently described *P*. *occultans* and *P*. *terminalis* [41] are not well defined and ambiguities remain for identification of these species. In agreement with Grünwald et al. [42], Kroon et al. [32] and Martin et al. [43, 44], a standard integrated approach including both morphology and molecular characterization (ITS and COI) was applied in this study to identify the new taxon from *Phytophthora* Clade 2 isolated from fruits and roots of pomelo in southern Vietnam. Cooke et al. [15] were the first to publish a database of ITS sequences (www.phytophthoradb.org) that covered all the known and available species of *Phytophthora* and, consequently, ITS has become a DNA barcode accepted by the scientific community for identification of *Phytophthora* at the species level [32, 16, 17, 45]. After the molecular revision of the taxonomy of *Phytophthora*, both *P*. *colocasiae* and *P*. *meadii* have been included as separate species in Clade 2, one of the largest clades in the *Phytophthora* phylogeny. Recently, however, it was shown that the ITS region is highly variable in *P*. *colocasiae*, suggesting that the ITS region alone may in some cases not be sufficient for species identification [46]. The DNA barcoding with cytochrome c oxidase subunit I (COI) is a relatively new approach [16] that, due to the faster evolution of COI as compared to ITS, in some cases can be more useful for discriminating recently diverged species than ITS.

In this study, the Clade 2 isolates obtained from symptomatic pomelo fruits, harvested directly from the tree, as well as from roots of pomelo trees, were clearly belonging to the same taxon on the basis of their ITS and COI sequences, thus fulfilling the currently accepted prerequisites for the identification of *Phytophthora* isolates at the species level [32, 25, 42, 44, 47, 48].

Morphologically, *P*. sp. mekongensis differs from *P*. *meadii* and *P*. *colocasiae* by being sterile while isolates of *P*. *meadii* are either homothallic or heterothallic and *P*. *colocasiae* is heterothallic [12]. In pathogenicity tests on fruits of various citrus species including pomelo and grapefruit, supposed to be a hybrid originating from pomelo as a parental species, isolates of *P*. sp. mekongensis caused typical symptoms of brown rot and were re-isolated from necrotic tissues of inoculated symptomatic fruits, thus fulfilling Koch’s postulates. In addition, in pathogenicity tests carried out in citrus seedlings, they induced gummosis of twigs and stems, a symptom commonly associated with infections of *P*. *citrophthora*, which is the most common species from Clade 2 responsible for important citrus diseases. Both geographic location and hosts may be relevant regarding the reports of *P*. *colocasiae* and *P*. *meadii*, as described in the Isolate Map Browser available on www.phytophthoradb.org [18]. In fact, according to Oudemans and Coffey [49], *P*. *meadii* has a high level of genetic similarity with *P*. *botryosa* and these two species probably represent two geographically separate populations that diverged from a common ancestor, since *P*. *meadii* was reported mainly from India and Sri Lanka while most records of *P*. *botryosa* are from Thailand, Malaysia, and Vietnam. Although partly outdated, this hypothesis is intriguing as minor differences in DNA sequences between *P*. *botryosa*, *P*. *citrophthora*, *P*. *colocasiae* and *P*. *meadii* could be interpreted as an ongoing speciation process resulting from geographic isolation or adaptation to either different host-plants or ecological conditions. In particular, *P*. *meadii* has been reported as a common pathogen of rubber tree (*Hevea brasiliensis*), which originates from Amazonia but is widely cultivated in southern Vietnam. Interestingly, ITS sequences of the new taxon *P*. sp. mekongensis from fruits and roots of pomelo in the Mekong River delta showed 100% similarity with the ITS sequence of a reference isolate of *P*. *meadii* (KC247914) sourced from rubber tree in Vietnam and deposited at GenBank (www.ncbi.nlm.nih.gov/). Unfortunately, this sequence has not been published in any scientific paper and the corresponding COI sequence is not available; therefore, it was not possible to confirm the identity between the isolate from rubber tree and the *P*. sp. mekongensis isolates from pomelo. This is the first report of Phytophthora brown rot of pomelo and the first time that a *Phytophthora* Clade 2 species other than *P*. *citricola* and *P*. *citrophthora* has been reported as a pathogen of citrus worldwide. Recently, *P*. *meadii* has been detected as a phylotype in the soil and tree rhizosphere of a citrus grove in southern Italy using an amplicon metagenomics approach based on genus specific primers [38, 50]. However, its pathogenicity on citrus was not demonstrated as the authors did not attempt to obtain living cultures. It is noteworthy that *P*. *meadii* is in the prioritization list of *Phytophthora* species of concern to the United States [44], a country with intense trade relations with Vietnam. Pathogenicity tests indicate that *P*. sp. mekongensis from pomelo fruits may infect other *Citrus* species and induce both fruit brown rot and gummosis of twigs and stems. Mixed infections of different *Phytophthora* species on the same host plant or plant organ are not unusual [51, 52, 53, 54] and their number is expected to increase rapidly with more frequent applications of molecular diagnostic techniques based on metagenomic approaches [38, 39, 55, 56]. In mixed infections, the incidence of each species as well as the isolation frequency depend on several factors, including virulence, host specialization, sporulation capacity and environmental conditions.

*Phytophthora insolita sensu lato* (*s*.*l*.), a high-temperature tolerant species within Clade 9 like *P*. *macilentosa*, *P*. *aquimorbida*, *P*. *hydrogena*, *P*. *irrigata*, *P*. *parsiana*, *P*. *virginiana* and *P*. *polonica* [57, 58], has been found occasionally in soils from citrus orchards in Taiwan and India [59, 60, 61]. It was originally described by Ann and Ko [59] as a homothallic species producing peculiar oogonia without antheridia, that can be barely distinguished from chlamydospores by the thickness of the wall. To our knowledge, this is the first report of *P*. *insolita s*.*l*. from Vietnam and the first report of this species causing brown rot of citrus fruits in the world. *P*. *insolita* is a common soil inhabitant of citrus groves in south-east Asia and the results from this study indicate that it is also a mild pathogen colonizing fibrous roots and citrus fruits lying on the ground. This species induced symptoms of brown rot on artificially inoculated fruits, confirming previous results of other authors [59], but it was never recovered from symptomatic fruits picked directly from the trees and did not induce gummosis on artificially inoculated twigs and stems. Therefore, it could be hypothesized that *P*. sp. mekongensis species isolated in Vietnam from pomelo fruits, being the prevalent and most virulent species, was very probably the primary causal agent of brown rot, while *P*. sp. prodigiosa, although quite common as a soil inhabitant in citrus groves in the tropics, colonized the fruits fallen to the ground as a secondary opportunistic pathogen. An additional hypothesis is that the two new taxa differ in their epidemiology. In fact, *P*. sp. mekongensis produces caducous sporangia which can be dispersed by rain splash enabling the spores to reach the fruits on the canopy. Conversely, *P*. sp. prodigiosa is characterized only by persistent sporangia, suggesting its nature as soilborne *Phytophthora* species, thus could explain why it was isolated only from fruits fallen to the ground. The epidemiological and economic relevance of *P*. sp. mekongensis as causal agent of citrus diseases in commercial citrus groves in Vietnam, its phylogenetic relationship to *P*. *citrophthora* and other pathogenic species in Clade 2 as well as regulatory policies for prevention of its introduction and spread in other citrus-producing areas and countries deserve further investigation. In conclusion, beyond the practical importance of the case study, it is noteworthy that citrus are among the most important host plants of Clade 2 *Phytophthora* species and a new taxon of this Clade has been reported as a pathogen of an ancestral Citrus species such as pomelo in South East Asia which has been indicated as a putative secondary diversification center of pomelo and other Citrus species [13]. Results of this study confirm that *P*. *insolita* s. l. is a common inhabitant of Citrus soils in Asia although it is a weak pathogen. As isolates related to *P*. *insolita* sourced in Vietnam appeared genetically distinct from those ones originating from other regions they are informally referred to as *P*. sp. prodigiosa. Interestingly isolates of this taxon from Vietnam did not form sexual structures, suggesting that both the definition and description of *P*. *insolita* are updated and deserve to be revised by examining a larger collection of isolates from different geographic areas. Overall results of this study corroborate previous findings [6, 13, 33, 30] indicating that Vietnam is a still unexplored reservoir of *Phytophthora* diversity.

## Supporting information

S1 Table Complete list of isolates of *Phytophthora* species sampled in the Mekong River Delta (Vietnam). (DOCX)

S2 Table List of isolates and GenBank accession numbers used in phylogenetic analysis. (DOCX)

## References

[1] Barrett HC, Rhodes AM. A numerical taxonomic study of affinity relationships in cultivated Citrus and its close relatives. Syst. Bot. 1976; 1: 105–136.

[2] Paull RE, Duarte O. Tropical fruits Vol.2, 2^nd^ edition Crop production science in horticulture 24. CABI, Wallingford, England; 2012 pp. 381.

[3] Ferguson L, Sakovich N, Roose M. California Citrus Rootstocks. Division of Agriculture and Natural Resources, University of California, Oakland, CA. Publication 21477, 1990. p. 18.

[4] Timmer LW, Duncan LW. Citrus Health Management. APS Press, The American Phytopathological Society, St. Paul, MN, USA; 1999 pp. 197.

[5] Thanh DVT, Vien NV, Drenth A. Phytophthora Diseases in Vietnam In Drenth A. and Guest D.I. (eds): Diversity and Management of *Phytophthora* in Southeast Asia. Australian Centre for international Agricultural Research, Canberra, Australia; 2004 pp. 238.

[6] Tri MV, Hoa NV, Chau NM, Pane A, Faedda R, De Patrizio A, et al Decline of jackfruit (*Artocarpus heterophyllus*) incited by *Phytophthora palmivora* in Vietnam. Phytopathol Mediterr. 2015; 54: 9−14.

[7] Masago H, Yoshikawa M, Fukada M, Nakanishi N. Selective inhibition of *Pythium* spp. on a medium for direct isolation of *Phytophthora* spp. from soils and plants. Phytopathology. 1977; 67: 425–428.

[8] Brasier CM, Sanchez-Hernandez E, Kirk SA. *Phytophthora inundata* sp. nov., a part heterothallic pathogen of trees and shrubs in wet or flooded soils. Mycological Research. 2003; 107: 477–484. 12825521 10.1017/s0953756203007548

[9] Jung T, Stukely MJC, Hardy GEStJ, White D, Paap T, Dunstan WA, Burgess TI. Multiple new *Phytophthora* species from ITS Clade 6 associated with natural ecosystems in Australia: evolutionary and ecological implications. Persoonia. 2011; 26: 13–39. 22025801 10.3767/003158511X557577 PMC3160797

[10] Nechwatal J, Bakonyi J, Cacciola SO, Cooke DEL, Jung T, Nagy ZÁ, et al The morphology, behaviour and molecular phylogeny of *Phytophthora* taxon Salixsoil and its redesignation as *Phytophthora lacustris* sp. nov. Plant Pathol. 2013; 62: 355–369.

[11] Jung T, Blaschke H. Phytophthora root rot in declining forest trees. Phyton. 1996; 36 (3): 95–102.

[12] Erwin DC and Ribeiro OK. Phytophthora Diseases Worldwide. 2^nd^ edn. St Paul, MN, USA, APS Press; 1996 p. 562.

[13] Biasi A, Martin FN, Cacciola SO, Magnano di San Lio G, Grünwald NJ, Schena L. Genetic analysis of Phytophthora nicotianae populations from different hosts using microsatellite markers. 2016; 10.1094/PHYTO-11-15-0299-R 27111805

[14] Evoli M. Molecular analysis of genetic diversity of Phytophthora citrophthora using nuclear and mitochondrial markers. 2016. Ph.D. Thesis. http://hdl.handle.net/10447/163338

[15] Cooke DEL, Drenth A, Duncan JM, Wagels G, Brasier CM. A molecular phylogeny of *Phytophthora* and related Oomycetes. Fungal Genet Biol. 2000; 30: 17–30. 10.1006/fgbi.2000.1202 10955905

[16] Robideau GP, De Cock AWAM, Coffey MD, Voglmayr H, Brouwer H, Bala K, et al DNA barcoding of oomycetes with cytochrome c oxidase subunit I and internal transcribed spacer. Mol Ecol Resour. 2011; 11: 1002–1011. 10.1111/j.1755-0998.2011.03041.x 21689384 PMC3195333

[17] Blair JE, Coffey MD, Park S-Y, Geiser DM, Kang S. A multi-locus phylogeny for *Phytophthora* utilizing markers derived from complete pathogen genomes. Fungal Genet Biol. 2007; 45: 266–277. 10.1016/j.fgb.2007.10.010 18039586

[18] Park J, Park B, Veeraraghavan N, Jung K, Lee YH, Blair JE, et al *Phytophthora* Database: A forensic database supporting the identification and monitoring of *Phytophthora*. Plant Dis. 2008; 92: 966–972. 10.1094/PDIS-92-6-0966 30769728

[19] Tamura K, Stecher G, Peterson D, Filipski A, Kumar S. MEGA6: Molecular Evolutionary Genetics Analysis version 6.0. Mol Biol Evol. 2013; 30: 2725–2729. 10.1093/molbev/mst197 24132122 PMC3840312

[20] Hall BG. Building phylogenetic trees from molecular data with MEGA. Mol Biol Evol. 2013; 30: 1229–1235. 10.1093/molbev/mst012 23486614

[21] Tamura K, Nei M. Estimation of the number of nucleotide substitutions in the control region of mitochondrial DNA in humans and chimpanzees. Mol Biol Evol. 1993; 10: 512–526. 8336541 10.1093/oxfordjournals.molbev.a040023

[22] Scanu B, Webber JF. Dieback and mortality of *Nothofagus* in Britain: ecology, pathogenicity and sporulation potential of the causal agent *Phytophthora pseudosyringae* Plant Pathol. 2016; 65: 26–36.

[23] Graham JH, Menge JA. Phytophthora-induced Diseases Pp. 12–15 in Compendium of Citrus Diseases. 2nd edition APS Press,St Paul, MN; 2000. p.92.

[24] Stamps DJ, Waterhouse GM, Newhook FJ, Hall GS. Revised Tabular Key to the Species of *Phytophthora*. Mycological papers, CAB International, Wallingford Oxon; 1990 162: pp. 1–28.

[25] Gallegly ME, Hong CX. *Phytophthora*: Identifying Species by Morphology and DNA Fingerprints. APS Press, St Paul, MN; 2008 p.158.

[26] Mammella MA, Cacciola SO, Martin F, Schena L. Genetic characterization of *Phytophthora nicotianae* by the analysis of polymorphic regions of the mitochondrial DNA. Fungal Biol. 2011; 115: 432–442. 10.1016/j.funbio.2011.02.018 21530925

[27] Mammella MA, Martin FN, Cacciola SO, Coffey MD, Faedda R, Schena L. Analyses of the population structure in a global collection of *Phytophthora nicotianae* isolates inferred from mitochondrial and nuclear DNA sequences. Phytopathology. 2013; 103: 610–622. 10.1094/PHYTO-10-12-0263-R 23384862

[28] Jung T, Orlikowski L, Henricot B, Abad-Campos P, Aday AG, Aguín Casal O., et al Widespread *Phytophthora* infestations in European nurseries put forest, semi-natural and horticultural ecosystems at high risk of Phytophthora diseases. Forest Pathol. 2016; 46: 134–163,

[29] Agosteo GE, Cacciola SO, Cavallaro P, Magnano di San Lio G, Russo MT. Bergamot Diseases. *Citrus bergamia* Bergamot and Its Derivatives. CRC Press, Taylor Francis Group, Boca Raton, FL, USA; 2014 pp. 37–48.

[30] Drenth A, Guest DI. Diversity and Management of *Phytophthora* in Southeast Asia. Australian Centre for international Agricultural Research, Canberra, Australia; 2004 p.238.

[31] Cacciola SO, Magnano di San Lio G. Management of citrus diseases caused by *Phytophthora* spp. In Ciancio A. and Mukerji K. G. (eds.) Integrated Management of Diseases Caused by Fungi, Phytoplasma and Bacteria. Series: Integrated Management of Plant Pests and Diseases. Springer Science +Business Media B. V., Heidelberg, Germany; 2008 vol. 3: pp. 61–84.

[32] Kroon LP, Brouwer H, de Cock AW, Govers F. The Genus *Phytophthora*. Phytopathology. 2012; 102: 348–364. 10.1094/PHYTO-01-11-0025 22185336

[33] De Patrizio A, Cacciola SO, Olsson C, Faedda R, Ramstedt M, Wright S, et al Identification of *Phytophthora* species infecting citrus in Vietnam. J Plant Pathol. 2013; 95: S4: S41.

[34] Chee KH. Variability of *Phytophthora* species from *Hevea brasiliensis*. T Brit Mycol Soc. 1969; 52: 425–436.

[35] Miyasaka SC, Lamour K, Shintaku M, Shrestha S, Uchida J. Taro leaf blight caused by *Phytophthora colocasiae* CABI, Boston, MA, USA In Lamour K. (ed.) *Phytophthora* a global perspective: 2013 pp. 104–112.

[36] Ko WH. Mating-type distribution of *Phytophthora colocasiae* on the island of Hawaii. Mycologia. 1979; 71: 434–437.

[37] Vavilov NI. The Origin, Variation, Immunity and Breeding of Cultivated Plants. (Translated from Russian by Chester K. S.) New York: Ronald Press Co; 1951.

[38] Prigigallo MI, Mosca S, Cacciola SO, Cooke DEL, Schena L. Molecular analysis of *Phytophthora* diversity in nursery-grown ornamental and fruit plants. Plant Pathol. 2015;. 64: 1308–1319.

[39] Prigigallo MI, Abdelfattah A, Cacciola SO, Faedda R, Sanzani SM, Cooke DEL, Schena L. Metabarcoding analysis of *Phytophthora* diversity using genus specific primers and 454 pyrosequencing. Phytopathology. 2015; 10.1094/PHYTO-07-15-0167-R 26574783

[40] Stamps DJ. Phytophthora meadii. IMI Descriptions of Fungi and Bacteria. 1985; 84: 834.

[41] Man In 't Veld WA, Rosendahl KC, van Rijswick PC, Meffert JP, Westenberg M, van de Vossenberg BT. et al *Phytophthora terminalis* sp. nov. and *Phytophthora occultans* sp. nov., two invasive pathogens of ornamental plants in Europe. Mycologia. 2015; 107 (1): 54–65. 10.3852/12-371 25261495

[42] Grünwald NJ, Martin FN, Larsen MM, Sullivan CM, Press CM, Coffey MD, Hansen EM. Phytophthora-ID.org: A sequence-based *Phytophthora* identification tool. Plant Dis. 2011; 95: 337–342. 10.1094/PDIS-08-10-0609 30743500

[43] Martin FN, Tooley PW. Phylogenetic relationships among *Phytophthora* species inferred from sequence analysis of mitochondrially encoded cytochrome oxidase I and II genes. Mycologia. 2003; 95: 269–284. 21156613

[44] Martin FN, Abad ZG, Balci Y, Ivors K. Identification and detection of *Phytophthora*: reviewing our progress, identifying our needs. Plant Dis. 2012; 96: 1080–1103. 10.1094/PDIS-12-11-1036-FE 30727075

[45] Abad ZG, Abad JA, Cacciola SO, Pane A, Faedda R, Moralejo E. et al *Phytophthora niederhauserii* sp. nov., a polyphagous species associated with ornamentals, fruit trees and native plants in 13 countries. Mycologia. 2014; 106: 431–447. 10.3852/12-119 24871599

[46] Nath VS, Hegde VM, Jeeva ML, Misra RS, Veena SS, Raj M, et al Rapid and sensitive detection of *Phytophthora colocasiae* responsible for the taro leaf blight using conventional and real-time PCR assay. FEMS Microbiol Lett. 2014; 352: 174–183. 10.1111/1574-6968.12395 24612149

[47] Bandyopadhyay R, Sharma K, Onyeka TJ, Aregbesola A, Kumar PL. First Report of Taro (*Colocasia esculenta*) Leaf Blight Caused by *Phytophthora colocasiae* in Nigeria. Plant Dis. 2011; 95: 618. 10.1094/PDIS-12-10-0890 30731969

[48] Omane E, Oduro KA, Cornelius EW, Opoku IY, Akrofi AY, Sharma K. et al First Report of Leaf Blight of Taro (*Colocasia esculenta*) caused by *Phytophthora colocasiae* in Ghana. Plant Dis. 2012; 96: 292. 10.1094/PDIS-09-11-0789 30731838

[49] Oudemans P, Coffey MD. A revised systematics of twelve papillate *Phytophthora* species based on isozyme analysis. Mycol Res. 1991; 95: 1025–1046.

[50] Scibetta S, Schena L, Chimento A, Cacciola SO, Cooke DEL. A molecular method to assess *Phytophthora* diversity in environmental samples. J Microbiol Meth. 2012; 88: 356–368. 10.1016/j.mimet.2011.12.012 22226752

[51] Pane A, Li Destri Nicosia MG, Cacciola SO. First report of *Phytophthora citrophthora* causing fruit brown rot of Feijoa in Italy. Plant Dis. 2001; 85: 97. 10.1094/PDIS.2001.85.1.97A 30832086

[52] Pane A, Cacciola SO, Chimento A, Allatta C, Scibetta S, Magnano di San Lio G. First report of *Phytophthor*a spp. as pathogens of *Pandorea jasminoides* in Italy. Plant Dis. 2008; 92: 313. 10.1094/PDIS-92-2-0313B 30769396

[53] Pane A, Cacciola SO, Scibetta S, Bentivenga G, Magnano di San Lio G. Four *Phytophthora* species causing foot and root rot of apricot in Italy. Plant Dis. 2009; 93: 844. 10.1094/PDIS-93-8-0844C 30764354

[54] Cacciola SO, Pane A, Faedda R, Rizza C, Badalà F, Magnano di San Lio G. Bud and root rot of windmill palm (*Trachycarpus fortunei*) caused by simultaneous infections of *Phytophthora palmivora* and *P*. *nicotianae* in Sicily. Plant Dis. 2011; 95: 769. 10.1094/PDIS-11-10-0823 30731928

[55] Vettraino AM, Bonants P, Tomassini A, Bruni N, Vannini A. Pyrosequencing as a tool for the detection of *Phytophthora* species: error rate and risk of false Molecular Operational Taxonomic Units. Lett Appl Microbiol. 2012; 55: 390–396. 10.1111/j.1472-765x.2012.03310.x 25998830

[56] Català S, Pérez-Sierra A, Abad-Campos P. The use of genus-specific amplicon pyrosequencing to assess *Phytophthora* species diversity using eDNA from soil and water in northern Spain. PLoS ONE. 2015; 10(3): e0119311 10.1371/journal.pone.0119311 25775250 PMC4361056

[57] Yang X, Hong C. *Phytophthora virginiana* sp. nov., a high-temperature tolerant species from irrigation water in Virginia. Mycotaxon. 2013; 126: 167–176

[58] Yang X, Richardson PA, Hong C. *Phytophthora* ×*stagnum* nothosp. nov., a new hybrid from irrigation reservoirs at ornamental plant nurseries in Virginia. PLoS ONE. 2014; 9(7): e103450 10.1371/journal.pone.0103450 25072374 PMC4114803

[59] Ann PJ, Ko WH. *Phytophthora insolita*, a new species from Taiwan. Mycologia. 1980: 72(6): 1180–1185.

[60] Das AK, Kumar A, Nerkar S, Bawage S. First report of *Phytophthora insolita* in India. Australasian Plant Dis Notes. 2012; 7: 131–132.

[61] Bawage S, Nerkar S, Kumar A, Das A. Morphological and molecular description of *Phytophthora insolita* isolated from citrus orchard in India. Journal of Mycology. 2013;

