# Supplementary material for: Two previously unknown Phytophthora species associated with brown rot of Pomelo (Citrus grandis) fruits in Vietnam
Source: PLoS One. 2017 Feb 16;12(2):e0172085. doi: 10.1371/journal.pone.0172085 (PMC5313238; doi:10.1371/journal.pone.0172085)
Supplement: S1 Table — (DOCX) [file pone.0172085.s001.docx]

**S1 Table. Complete list of isolates of *Phytophthora* species sampled in the Mekong River Delta (Vietnam).**

|  |  |  |  |  |  |  |  |
| --- | --- | --- | --- | --- | --- | --- | --- |
| Isolate code | Representative isolate | *Phytophthora* spp. | Source | Host | Geographic origin (province) | ITS Accession Number | COI  Accession Number |
| PF6a2 | PF6a2 | *P.* sp. mekongensis | Fruit | Pomelo | Vĩnh Long | KC875838 | KT366920 |
| PF6a2/1 | PF6a2 | *P.* sp. mekongensis | Fruit | Pomelo | Vĩnh Long | KU363456 | KU363489 |
| PF6a2/2 | PF6a2 | *P.* sp. mekongensis | Fruit | Pomelo | Vĩnh Long | KU363457 | KU363490 |
| PF6a2/3 | PF6a2 | *P.* sp. mekongensis | Fruit | Pomelo | Vĩnh Long | KU363458 | KU363491 |
| PF6a2/4 | PF6a2 | *P.* sp. mekongensis | Fruit | Pomelo | Vĩnh Long | KU363459 | KU363492 |
| PF6a2/5 | PF6a2 | *P.* sp. mekongensis | Fruit | Pomelo | Vĩnh Long | KU363460 | KU363493 |
| PF6a2/6 | PF6a2 | *P.* sp. mekongensis | Fruit | Pomelo | Vĩnh Long | KU363461 | KU363494 |
| PF6a2/7 | PF6a2 | *P.* sp. mekongensis | Fruit | Pomelo | Vĩnh Long | KU363462 | KU363495 |
| PF6a2/8 | PF6a2 | *P.* sp. mekongensis | Fruit | Pomelo | Vĩnh Long | KU363463 | KU363496 |
| PF6a2/9 | PF6a2 | *P.* sp. mekongensis | Fruit | Pomelo | Vĩnh Long | KU363464 | KU363497 |
| PF6a2/10 | PF6a2 | *P.* sp. mekongensis | Fruit | Pomelo | Vĩnh Long | KU363465 | KU363498 |
| PF6b | PF6a2 | *P.* sp. mekongensis | Fruit | Pomelo | Vĩnh Long | KU363466 | KU363499 |
| PF6b/1 | PF6a2 | *P.* sp. mekongensis | Fruit | Pomelo | Vĩnh Long | KU363467 | KU363500 |
| PF6b/2 | PF6a2 | *P.* sp. mekongensis | Fruit | Pomelo | Vĩnh Long | KU363468 | KU363501 |
| PF6b/3 | PF6a2 | *P.* sp. mekongensis | Fruit | Pomelo | Vĩnh Long | KU363469 | KU363502 |
| PF6b/4 | PF6a2 | *P.* sp. mekongensis | Fruit | Pomelo | Vĩnh Long | KU363470 | KU363503 |
| PF6b/5 | PF6a2 | *P.* sp. mekongensis | Fruit | Pomelo | Vĩnh Long | KU363471 | KU363504 |
| PF6b/6 | PF6a2 | *P.* sp. mekongensis | Fruit | Pomelo | Vĩnh Long | KU363472 | KU363505 |
| PF6b/7 | PF6a2 | *P.* sp. mekongensis | Fruit | Pomelo | Vĩnh Long | KU363473 | KU363506 |
| PF6b/8 | PF6a2 | *P.* sp. mekongensis | Fruit | Pomelo | Vĩnh Long | KU363474 | KU363507 |
| PF6b/9 | PF6a2 | *P.* sp. mekongensis | Fruit | Pomelo | Vĩnh Long | KU363475 | KU363508 |
| PF6b/10 | PF6a2 | *P.* sp. mekongensis | Fruit | Pomelo | Vĩnh Long | KU363476 | KU363509 |
| PF6a | PF6a2 | *P.* sp. mekongensis | Fruit | Pomelo | Vĩnh Long | KU363477 | KU363510 |
| PF6a3 | PF6a2 | *P.* sp. mekongensis | Fruit | Pomelo | Vĩnh Long | KU363478 | KU363511 |
| PF6a3/1 | PF6a2 | *P.* sp. mekongensis | Fruit | Pomelo | Vĩnh Long | KU363479 | KU363512 |
| PF6a3/2 | PF6a2 | *P.* sp. mekongensis | Fruit | Pomelo | Vĩnh Long | KU363480 | KU363513 |
| PF6a3/3 | PF6a2 | *P.* sp. mekongensis | Fruit | Pomelo | Vĩnh Long | KU363481 | KU363514 |
| PF6a3/4 | PF6a2 | *P.* sp. mekongensis | Fruit | Pomelo | Vĩnh Long | KU363482 | KU363515 |
| PF6a3/5 | PF6a2 | *P.* sp. mekongensis | Fruit | Pomelo | Vĩnh Long | KU363483 | KU363516 |
| PF6a3/6 | PF6a2 | *P.* sp. mekongensis | Fruit | Pomelo | Vĩnh Long | KU363484 | KU363517 |
| PF6a3/7 | PF6a2 | *P.* sp. mekongensis | Fruit | Pomelo | Vĩnh Long | KU363485 | KU363518 |
| PF6a3/8 | PF6a2 | *P.* sp. mekongensis | Fruit | Pomelo | Vĩnh Long | KU363486 | KU363519 |
| PF6a3/9 | PF6a2 | *P.* sp. mekongensis | Fruit | Pomelo | Vĩnh Long | KU363487 | KU363520 |
| PF6a3/10 | PF6a2 | *P.* sp. mekongensis | Fruit | Pomelo | Vĩnh Long | KU363488 | KU363521 |
| PF6f2 | PF6f2 | *P.* sp. mekongensis | Fruit | Pomelo | Vĩnh Long | KC875839 | KT366920 |
| PF6f2/1 | PF6f2 | *P.* sp. mekongensis | Fruit | Pomelo | Vĩnh Long | KU363560 | KU363522 |
| PF6f2/2 | PF6f2 | *P.* sp. mekongensis | Fruit | Pomelo | Vĩnh Long | KU363561 | KU363523 |
| PF6f2/3 | PF6f2 | *P.* sp. mekongensis | Fruit | Pomelo | Vĩnh Long | KU363562 | KU363524 |
| PF6f2/4 | PF6f2 | *P.* sp. mekongensis | Fruit | Pomelo | Vĩnh Long | KU363563 | KU363525 |
| PF6f2/5 | PF6f2 | *P.* sp. mekongensis | Fruit | Pomelo | Vĩnh Long | KU363564 | KU363526 |
| PF6f2/6 | PF6f2 | *P.* sp. mekongensis | Fruit | Pomelo | Vĩnh Long | KU363565 | KU363527 |
| PF6f2/7 | PF6f2 | *P.* sp. mekongensis | Fruit | Pomelo | Vĩnh Long | KU363566 | KU363528 |
| PF6f2/8 | PF6f2 | *P.* sp. mekongensis | Fruit | Pomelo | Vĩnh Long | KU363567 | KU363529 |
| PF6f2/9 | PF6f2 | *P.* sp. mekongensis | Fruit | Pomelo | Vĩnh Long | KU363568 | KU363530 |
| PF6f2/10 | PF6f2 | *P.* sp. mekongensis | Fruit | Pomelo | Vĩnh Long | KU363569 | KU363531 |
| PF6f2/11 | PF6f2 | *P.* sp. mekongensis | Fruit | Pomelo | Vĩnh Long | KU363570 | KU363532 |
| PF6f2/12 | PF6f2 | *P.* sp. mekongensis | Fruit | Pomelo | Vĩnh Long | KU363571 | KU363533 |
| PF6f1 | PF6f2 | *P.* sp. mekongensis | Fruit | Pomelo | Vĩnh Long | KU363572 | KU363534 |
| PF6f1/1 | PF6f2 | *P.* sp. mekongensis | Fruit | Pomelo | Vĩnh Long | KU363573 | KU363535 |
| PF6f1/2 | PF6f2 | *P.* sp. mekongensis | Fruit | Pomelo | Vĩnh Long | KU363574 | KU363536 |
| PF6f1/3 | PF6f2 | *P.* sp. mekongensis | Fruit | Pomelo | Vĩnh Long | KU363575 | KU363537 |
| PF6f1/4 | PF6f2 | *P.* sp. mekongensis | Fruit | Pomelo | Vĩnh Long | KU363576 | KU363538 |
| PF6f1/5 | PF6f2 | *P.* sp. mekongensis | Fruit | Pomelo | Vĩnh Long | KU363577 | KU363539 |
| PF6f1/6 | PF6f2 | *P.* sp. mekongensis | Fruit | Pomelo | Vĩnh Long | KU363578 | KU363540 |
| PF6f1/7 | PF6f2 | *P.* sp. mekongensis | Fruit | Pomelo | Vĩnh Long | KU363579 | KU363541 |
| PF6f1/8 | PF6f2 | *P.* sp. mekongensis | Fruit | Pomelo | Vĩnh Long | KU363580 | KU363542 |
| PF6f1/9 | PF6f2 | *P.* sp. mekongensis | Fruit | Pomelo | Vĩnh Long | KU363581 | KU363543 |
| PF6f1/10 | PF6f2 | *P.* sp. mekongensis | Fruit | Pomelo | Vĩnh Long | KU363582 | KU363544 |
| PF6f1/11 | PF6f2 | *P.* sp. mekongensis | Fruit | Pomelo | Vĩnh Long | KU363583 | KU363545 |
| PF6f | PF6f2 | *P.* sp. mekongensis | Fruit | Pomelo | Vĩnh Long | KU363584 | KU363546 |
| PF6f/1 | PF6f2 | *P.* sp. mekongensis | Fruit | Pomelo | Vĩnh Long | KU363585 | KU363547 |
| PF6f/2 | PF6f2 | *P.* sp. mekongensis | Fruit | Pomelo | Vĩnh Long | KU363586 | KU363548 |
| PF6f/3 | PF6f2 | *P.* sp. mekongensis | Fruit | Pomelo | Vĩnh Long | KU363587 | KU363549 |
| PF6f/4 | PF6f2 | *P.* sp. mekongensis | Fruit | Pomelo | Vĩnh Long | KU363588 | KU363550 |
| PF6f/5 | PF6f2 | *P.* sp. mekongensis | Fruit | Pomelo | Vĩnh Long | KU363589 | KU363551 |
| PF6f/6 | PF6f2 | *P.* sp. mekongensis | Fruit | Pomelo | Vĩnh Long | KU363590 | KU363552 |
| PF6f/7 | PF6f2 | *P.* sp. mekongensis | Fruit | Pomelo | Vĩnh Long | KU363591 | KU363553 |
| PF6f/8 | PF6f2 | *P.* sp. mekongensis | Fruit | Pomelo | Vĩnh Long | KU363592 | KU363554 |
| PF6f/9 | PF6f2 | *P.* sp. mekongensis | Fruit | Pomelo | Vĩnh Long | KU363593 | KU363555 |
| Pr3 | Pr3 | *P.* sp. mekongensis | Root | Pomelo | Ben Tre | KM501564 | KU640394 |
| Pr3/1 | Pr3 | *P.* sp. mekongensis | Root | Pomelo | Ben Tre | KU363594 | KU363556 |
| Pr3/2 | Pr3 | *P.* sp. mekongensis | Root | Pomelo | Ben Tre | KU363595 | KU363557 |
| Pr3/3 | Pr3 | *P.* sp. mekongensis | Root | Pomelo | Ben Tre | KU363596 | KU363558 |
| Pr3/4 | Pr3 | *P.* sp. mekongensis | Root | Pomelo | Ben Tre | KU363597 | KU363559 |
| PF6e | PF6e | *P.* sp. prodigiosa | Fruit | Pomelo | Vĩnh Long | KC875840 | KT366918 |
| PF6e/1 | PF6e | *P.* sp. prodigiosa | Fruit | Pomelo | Vĩnh Long | KU363438 | KU363447 |
| PF6e/2 | PF6e | *P.* sp. prodigiosa | Fruit | Pomelo | Vĩnh Long | KU363439 | KU363448 |
| PF6e/3 | PF6e | *P.* sp. prodigiosa | Fruit | Pomelo | Vĩnh Long | KU363440 | KU363449 |
| PF6d | PF6e | *P.* sp. prodigiosa | Fruit | Pomelo | Vĩnh Long | KU363441 | KU363450 |
| PF6d/1 | PF6e | *P.* sp. prodigiosa | Fruit | Pomelo | Vĩnh Long | KU363442 | KU363451 |
| PF6d/2 | PF6e | *P.* sp. prodigiosa | Fruit | Pomelo | Vĩnh Long | KU363443 | KU363452 |
| PF6c | PF6e | *P.* sp. prodigiosa | Fruit | Pomelo | Vĩnh Long | KU363444 | KU363453 |
| PF6c/1 | PF6e | *P.* sp. prodigiosa | Fruit | Pomelo | Vĩnh Long | KU363445 | KU363454 |
| PF6c/1 | PF6e | *P.* sp. prodigiosa | Fruit | Pomelo | Vĩnh Long | KU363446 | KU363455 |
| Pr1 | Pr1 | *P.* sp. prodigiosa | Root | Mandarin/ Volkamer lemon | Dong Thap | KM501564 | KU363433 |
| Pr1/1 | Pr1 | *P.* sp. prodigiosa | Root | Mandarin/ Volkamer lemon | Dong Thap | KU363429 | KU363434 |
| Pr1/2 | Pr1 | *P.* sp. prodigiosa | Root | Mandarin/ Volkamer lemon | Dong Thap | KU363430 | KU363435 |
| Pr1/3 | Pr1 | *P.* sp. prodigiosa | Root | Mandarin/ Volkamer lemon | Dong Thap | KU363431 | KU363436 |
| Pr1/4 | Pr1 | *P.* sp. prodigiosa | Root | Mandarin/ Volkamer lemon | Dong Thap | KU363432 | KU363437 |
