# Supplementary material for: Two previously unknown Phytophthora species associated with brown rot of Pomelo (Citrus grandis) fruits in Vietnam
Source: PLoS One. 2017 Feb 16;12(2):e0172085. doi: 10.1371/journal.pone.0172085 (PMC5313238; doi:10.1371/journal.pone.0172085)
Supplement: S2 Table — (DOCX) [file pone.0172085.s002.docx]

**S2 Table. List of isolates and GenBank accession numbers used in phylogenetic analysis**.

| Species | Isolate name | Clade | COI GenBank  accession numbers | ITS GenBank  accession numbers |
| --- | --- | --- | --- | --- |
| *P.* sp. mekongensis *^1^* | PF6a2 | *2* | KT366920 | KC875838 |
| *P.* sp. mekongensis *^1^* | PF6f2 | *2* | KT366919 | KC875839 |
| *P.* sp. mekongensis *^1^* | Pr3 | *2* | KU640394 | KM501564 |
| *P.* aff. *meadii* | CBS 235.30 | 2 | HQ708214 | HQ643140 |
| *P.* aff. *meadii* | CBS 238.28 | 2 | HQ708213 | HQ643139 |
| *P.*  *meadii* | p75 | 2 | GU945489 | GU993903 |
| *P.* *meadii* | CBS 219.88 | 2 | HQ708324 | HQ643268 |
| *P. meadii* | P6128 | 2 | HQ261354 | HQ261607 |
| *P.* *colocasiae* | P6290 | 2 | HQ261287 | HQ261540 |
| *P.* *colocasiae* | P6317 | 2 | HQ261286 | HQ261539 |
| *P. botryosa* | P1044 | 2 | HQ261257 | HQ261510 |
| *P. botryosa* | P3425 | 2 | HQ261256 | HQ261509 |
| *P. botryosa* | p44 | 2 | GU945466 | GU993883 |
| *P. botryosa* | P6944 | 2 | HQ261255 | HQ261508 |
| *P. botryosa* | P6945 | 2 | HQ261254 | HQ261507 |
| *P. botryosa** | CBS 581.69 | 2 | HQ708222 | HQ643151 |
| *P. citrophthora* | 136 | 2 | GU945471 | GU993889 |
| *P. citrophthora* | CBS 111338 | 2 | HQ708275 | HQ643208 |
| *P. citrophthora* | CBS 111339 | 2 | HQ708274 | HQ643207 |
| *P. citrophthora* | CBS 111726 | 2 | HQ708273 | HQ643206 |
| *P. citrophthora* | P10368 | 2 | HQ261282 | HQ261535 |
| *P. citrophthora* | CBS 950.87 | 2 | HQ708272 | HQ643205 |
| *P. citrophthora* | P10341 | 2 | HQ261283 | HQ261536 |
| *P.* sp. prodigiosa^1^ | PF6e | 9 | KT366918 | KC875840 |
| *P.* sp. prodigiosa^1^ | Pr1 | 9 | KU363433 | KM501564 |
| *P. insolita* | IMI 288805 | 9 | GU945482 | GU993897 |
| *P. insolita* | P6195 | 9 | HQ261338 | HQ261591 |
| *P. insolita* | P6703 | 9 | HQ261337 | HQ261590 |
| *P. captiosa* | P10720 | 9 | HQ261268 | HQ261521 |
| *P. captiosa* | P10719 | 9 | HQ261269 | HQ261522 |
| *P. fallax* | P10722 | 9 | HQ261306 | HQ261559 |
| *P. fallax* | P10723 | 9 | HQ261305 | HQ261558 |
| *P. fallax* | P10725 | 9 | HQ261304 | HQ261557 |
| *P.*  *parsiana* | P15164 | 9 | HQ261386 | HQ261639 |
| *P.*  *parsiana* | P21281 | 9 | HQ261385 | HQ261638 |
| *P.*  *parsiana* | P21282 | 9 | HQ261384 | HQ261637 |
| *P.*  *polonica* | P15004 | 9 | HQ261394 | HQ261647 |
| *P.*  *polonica* | P15005 | 9 | HQ261393 | HQ261646 |
| *P.*  *quininea* | P8488 | 9 | HQ261407 | HQ261660 |
| *P.*  *quininea* | P3247 | 9 | HQ261408 | HQ261661 |
| *P.*  *quininea** | CBS 407.48 | 9 | HQ708386 | HQ643338 |
| *P.*  *macrochlamydospora* | IMI 351473 | 9 | GU945488 | GU993902 |
| *P.*  *macrochlamydospora* | P10267 | 9 | HQ261353 | HQ261606 |
| *P.*  *macrochlamydospora* | P8017 | 9 | HQ261352 | HQ261605 |

^1^= Vietnamese isolates.

*= ex-type isolate.
